# Exploring burnout and the association with the educational climate in pediatric residents in Thailand

**DOI:** 10.1186/s12909-019-1687-7

**Published:** 2019-07-05

**Authors:** Pongtong Puranitee, Fred F. C. J. Stevens, Samart Pakakasama, Adisak Plitponkarnpim, Sakda Arj-OngVallibhakara, Jamiu O. Busari, Sylvia Heeneman, Walther N. K. A. van Mook

**Affiliations:** 1 Faculty of Medicine Ramathibodi Hospital, Mahidol University, 270 Rama VI Road, Rajthevi, Bangkok 10400 Thailand; 2 Department of Educational Development & Research, Faculty of Health, Medicine & Life Sciences (FHML), Maastricht University (UM), Maastricht, The Netherlands; 3 Section for Clinical Epidemiology and Biostatistics, Faculty of Medicine Ramathibodi Hospital, Mahidol University, 270 Rama VI Road, Rajthevi, Bangkok 10400 Thailand; 4 Child Safety Promotion and Injury Prevention and Research Center, Faculty of Medicine Ramathibodi Hospital, Mahidol University, 270 Rama VI Road, Rajthevi, Bangkok 10400 Thailand; 5 Department of Pathology, Faculty of Health, Medicine & Life Sciences (FHML), School of Health Profession Education, Maastricht University (UM), Maastricht, The Netherlands; 6 Department of Intensive Care Medicine, Maastricht University Medical Centre, Maastricht, The Netherlands

**Keywords:** Burnout, Learning climate, Medical education, Pediatric resident

## Abstract

**Background:**

This study, undertaken in Bangkok, Thailand, explored the extent to which paediatric residents in a non-Western setting experienced burnout and the potential association with factors in the medical educational climate and work-related quality of life.

**Methods:**

An exploratory sequential mixed methods design was employed in a cross-sectional study. The initial, quantitative phase used the validated Maslach Burnout Inventory, the Postgraduate Hospital Educational Environmental Measure (PHEEM) and Work-Related Quality of Life scale (WRQoL). Regression analysis was used to identify the correlation between burnout and educational climate. Thereafter, residents in all years with high levels of burnout on subscales were interviewed individually.

**Results:**

Forty-one paediatric residents completed the three questionnaires. None had high levels related to burnout in *all three* domains (emotional exhaustion, high level of depersonalization and perceived low personal accomplishment), seven (17%) showed high levels in two out of three domains. Emotional exhaustion and educational climate (perceptions of role autonomy, perceptions of teaching, perceptions of social support) were correlated with work-related quality of life. In the interviews, the main themes related to burnout were inappropriate tasks, teachers and teaching styles, the perception of knowledge insecurity relating to task performance, time dimensions, life crisis during training, role expectations and work allocation clarity, and facilities such as accommodation.

**Conclusions:**

The study, in a non-Western setting, demonstrated a positive relation between educational climate and work-related quality of life. To help reduce the risk of burnout, the following factors were identified: minimize unnecessary or duplicated workload, schedule time arrangements to avoid extension of regular duty hours, and clearly define role expectations. The impact of inappropriate tasks, teachers and teaching styles (including unsafe environment) on the incidence of burnout was also highlighted. Additional studies focusing on teaching styles, safe learning climate and mistreatment in a non-Western context are needed.

## Background

Burnout is a psychological condition presenting a response to chronic interpersonal stressors on the job. Burnout, as defined by Maslach (1993), is a triad of high exhaustion, high depersonalization and lack of personal accomplishment [1]. Research among physicians revealed differences in burnout prevalence across (sub) specialties. A periodic survey of American Academy of Pediatrics (AAP) members (*n* = 1616; response rate 63%), showed that 22% of the respondents indicated to be experiencing burnout, while 45% had experienced burnout in the past [2]. From 2011 to 2014, general pediatrics in U.S. was found to be one of the ten subspecialties that experienced a more than 10% increase in burnout (35% vs. 46%) [3]. Burnout, highly prevalent among pediatricians, has been identified as a problem among pediatric residents and could ultimately have consequences for patient care. A study among 258 pediatric residents of 11 New England Pediatric Residency Consortium programs in U.S. revealed that residents experiencing burnout had significantly greater odds of displaying suboptimal patient care attitudes and behaviors, leading to maltreatment or medical errors, or ignoring the social impact of illness [4].

High levels of burnout amongst residents across cultures and countries have uniformly been described, although some differences between countries can be observed. In Brazil, 53% of pediatric residents experienced burnout [5], a percentage similar reported in a survey in Canada, where Nolan et al. found 42% of pediatric residents met criteria of burnout [6]. In North America, Simpkin et al. reported that 31% of pediatric residents had burnout and determined high levels of stress from uncertainty and low levels of resilience as factors strongly related to burnout [7]. A longitudinal prospective study of pediatric residents in North America showed that the prevalence of burnout increased from 2 to 24% between the start of residency and mid-intern year, without significant change over a 2-year period [8]. In the U.S., Olson et al. showed that approximately 40% of first year pediatric residents experienced burnout. Self-compassion and mindfulness were factors inversely associated with burnout [9]. In summary, the prevalence of burnout among residents thus varies across countries and factors related to it might at least partly originate from the workplace.

**Fig. 1:**
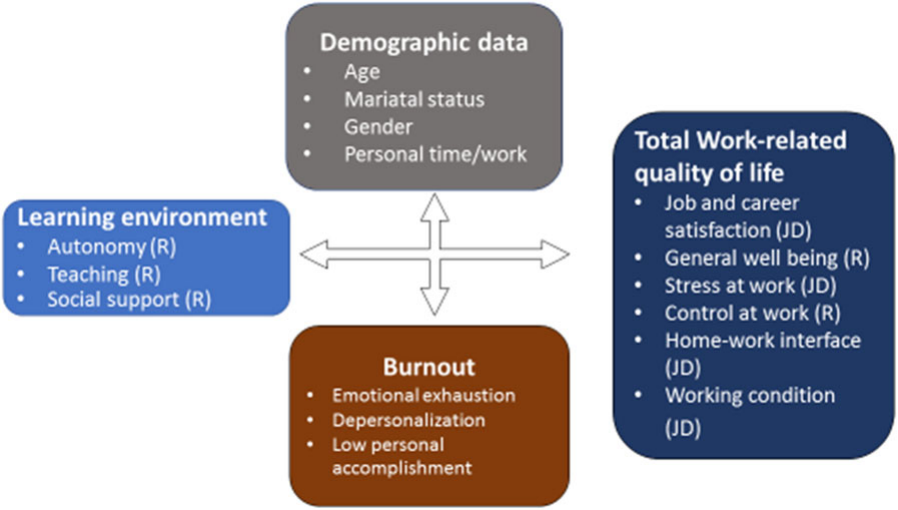
The conceptual framework formulated for this study related to the job demands and resources model

Work-related quality of life is more or less similar to employee well-being and includes work-based factors such as job satisfaction, as well as broader non-work factors such as general life satisfaction, feelings of well-being [10] and work-related stress [11, 12]. A study done in the U.S., showed that factors contributing to first year pediatric resident burnout included time demands, lack of control, difficulties in work planning and work organization, difficult job situations and interpersonal relationship problems [10]. A separate study on burnout, showed a negative correlation between job satisfaction and burnout levels among Turkish pediatric residents [13]. In summary, factors such as job satisfaction, stress and control at work, working condition, home-work interface have been associated with the incidence of burnout in pediatric residents in Western countries [10–13]. In non-Western countries such as Japan for example, factors contributing to burnout include high workload, stress intolerance, interpersonal difficulties, and generation gaps regarding work-life balance [14]. In Thailand, Srikamet et al. conducted a survey on job burnout and related factors among residents in all years across subspecialties. They reported that residents trained in *larger* subspecialties (such as pediatrics, internal medicine, surgery) had a significantly higher prevalence of burnout, compared to residents in *smaller* subspecialties (such as ear-nose-throat, ophthalmology). The differences between the larger and smaller subspecialties that might be related to burnout were higher workloads, longer working/duty hours and specific characteristic of the learning environment of  *larger* subspecialties [15]. Summarizing, findings in the literature suggest that learning in an environment that is considered to be demanding is associated with higher levels of burnout. This finding aligns with the conceptual framework of the job demands-resources model (JD-R model, Fig. 1). “Job demand” is a physical, psychological, social, or organizational aspect of the job that requires effort, while “resources” are aspects of the job that are functional in achieving work goals; decrease job demands or stimulate individual growth, learning, and development [16–18]. High levels of job demand with lack of resources therefore lead to a decrease in work engagement [19].

The educational climate is a crucial factor for residents and tends to impact learning achievement as well as residents wellness. The educational climate has a significant correlation with residents burnout [20, 21], especially in an intensely demanding workplace where a higher number of work shifts has been associated with a higher level of burnout [22, 23].

**Fig. 2:**
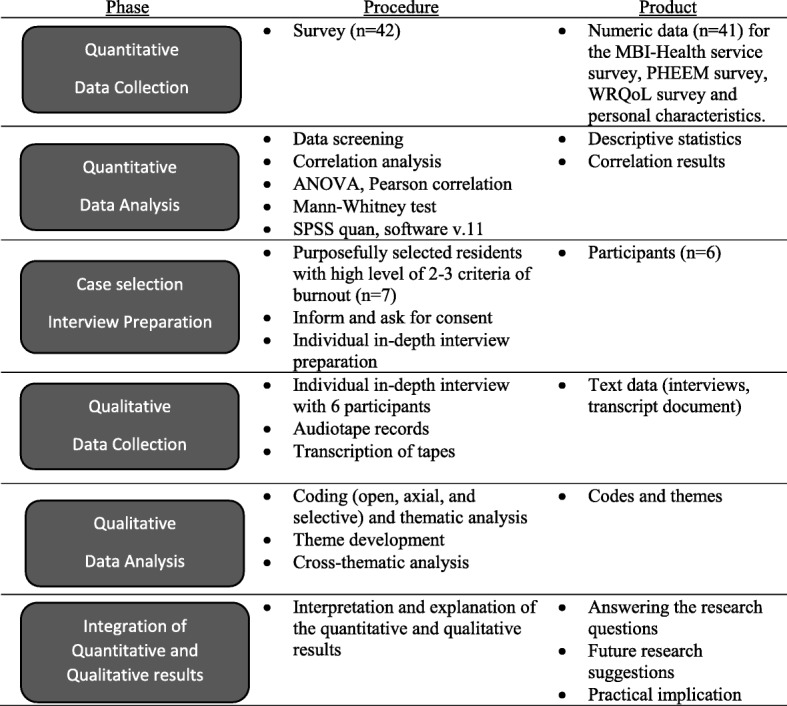
Visual model of the sequential explanatory mixed method study design

In summary, burnout is a highly prevalent problem among pediatric residents worldwide, with adverse consequences for patient care. Main factors related to burnout in Western countries were job satisfaction, stress at work, control at work, as well as the educational climate. However, as far as we know, factors related to pediatric residents burnout especially regarding educational climate and work quality of life have not yet been studied in non-Western contexts. Therefore, this study explored the prevalence of burnout among pediatric residents, and factors related to burnout in the Thai context, in particular aspects of the educational climate, and work related quality of life. The study addresses the following research questions (Fig. 2):

1. To what extent do paediatric residents in a non-Western setting experience burnout? (Quantitative approach)
2. What is the relation between burnout and personal characteristics of residents’ learning environment and work-related quality of life? (Quantitative approach)
3. What factors in the medical educational environment and work-related quality of life relate to burnout? (Quantitative and qualitative approach)

## Methods

The study was conducted in the Paediatric Department, Ramathibodi Hospital, Bangkok, Thailand in 2016. An exploratory mixed-methods approach was employed, consisting of sequentially collecting and respectively analyzing quantitative and qualitative data (Fig. 2). The choice for a mixed methods approach was based on its ability to provide more comprehensive and in depth details to answer the research questions.

### Quantitative phase, surveys

The first phase employed a quantitative cross-sectional survey which included background variables, measurements of burnout, the perceived learning environment, and work-related quality of life. The following background-characteristics were measured: age, gender, year of training, marital status, responsibility for the family’s or own financial burden, sleep hours/night, study time hours/week, non-academic leisure time hours/week.

Burnout was measured with the Maslach Burnout Inventory (MBI) - Health service survey, an internationally acknowledged and validated instrument [1]. The MBI is a standard 22-item questionnaire (7-point Likert scale). The instrument consists of three sections which measures levels of a) emotional exhaustion, b) depersonalisation and c) personal accomplishment [15]. The MBI has previously been translated into Thai and tested for reliability. Cronbach’s alpha coefficients for the sections were 0.92, 0.66 and 0.65, respectively [15].

Learning environment was measured with the Postgraduate Hospital Educational Environmental Measure (PHEEM) questionnaire. This questionnaire consists of 40 items, divided into three sections: a) autonomy, b) teaching, and c) social support (5-point Likert scale, ranging from 4 = strongly agree to 0 = strongly disagree). The original instrument, tested in the UK, had a Cronbach’s alpha reliability of 0.91 [24]. Translation into the Thai language was done by an experienced translator in the medical field and then back-translated to English by another experienced translator, leading to further refinement into the Thai language. The original English questionnaire was then analyzed for degree of agreement with the back-translation and rated by three faculty medical instructors from the paediatric department. Kappa’s agreement scores ranged between 0.61–0.80 and were confirmed as substantial at 80–95% of agreement.

Work-related Quality of life was measured with the Work-Related Quality of Life scale (WRQoL), a 23-item psychometric scale divided into six subscales of factors contributing to quality of life: Job and Career Satisfaction (JCS), General Well-Being (GWB), Stress at Work (SAW), Control at Work (CAW), Home-Work Interface (HWI) and Working Conditions (WCS). The questionnaire is composed of 5-point Likert scale items (1 = strongly disagree; 5 = strongly agree). Its reliability (Cronbach’s alpha of 0.75–0.86) were verified among medical personnel in the United Kingdom [12]. We used the Thai-translated and validated 34item WRQoL version 2 with 7 sub-scales (Job and Career Satisfaction (JCS), General Well-Being (GWB), Stress at Work (SAW), Control at Work (CAW), Home-Work Interface (HWI) and Working Conditions (WCS) and employee engagement (EET)), which had a content validity index of the scale of 0.97. Overall Cronbach’s alpha was 0.93 with good test-retest reliabilityof0.892 [25].

The three surveys (110 items) were distributed in written format among all 42 pediatric residents across all years of training, also inviting them to later participate in interviews.

### Qualitative phase, individual interviews

Purposeful sampling was used, participation was voluntary and withdrawal was possible at any given moment. Residents in all 3 years with a high score in at least two of the three subscales of the MBI questionnaire, and with varying scores on the other questionnaires, were invited (*n* = 7) in order to further explore the perceived influence of educational climate and its relation to burnout. Six residents responded positively and were interviewed individually. The interviews were conducted by PT, who is a supervising physician and the conversations were audio-taped. The interview was semi-structured around general open-ended questions concerning two main topics: a) characteristics in the medical educational environment related to burnout risk, and b) What could be improved in the medical educational environment to reduce the risk of burnout.

### Analysis

#### Quantitative data

The association between categorical data of burnout level and baseline characteristics was calculated using Phi coefficient. To identify the relations between levels of burnout, PHEEM, work-related quality of life and other variables, hierarchical single regression analyses were conducted. Pearson zero-order and Spearman rank-order correlations were used to identify the relation between PHEEM (and subscales) and work-related quality of life.

#### Qualitative data

Literal transcripts from the interviews were analyzed by two researchers independently. The researchers familiarized with the data by listening to the audio-tapes and reading the full data sets several times. The interviews were coded using an inductive approach. The main findings were verified with the participants in a process of member checking. Any discrepancies in coding were discussed in the research team until consensus was reached [26]. Thematic analysis was done by hand and then translated into English. Themes emerged from the independently iterative analysis and then presented as results.

### Ethical approval

The study was reviewed and approved by the Institutional Review Board of the Faculty of Medicine, Ramathibodi Hospital, Mahidol University (IRB no.08–58-42). All participants were informed, both verbally and in writing and consented to complete the questionnaire and interview. Since burnout is a sensitive issue, participants had the option to terminate project participation at any stage.

## Results

### Quantitative part, surveys

**Fig. 3:**
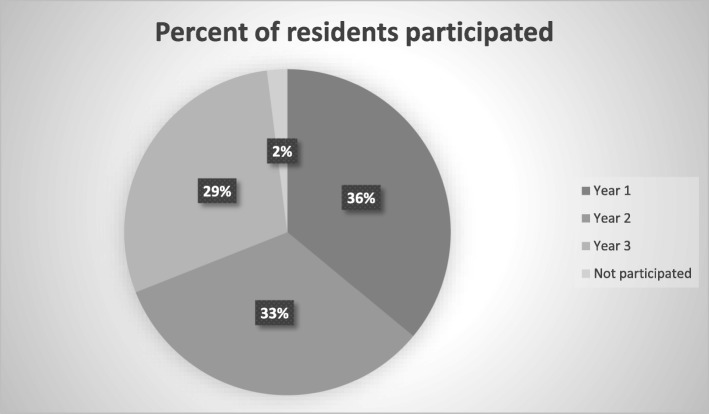
Distribution of residents participated in the study in all year

Forty-two paediatric residents were invited to participate with a 97% response rate (one refused to participate); 75% of respondents were female. The distribution of responding residents over years 1, 2 and 3 was 15 (36%), 14 (33%) and 12 (29%) respectively (Fig. 3).

The MBI questionnaire identified 7 residents (17%) perceiving high emotional exhaustion, five (12%) showed high depersonalization and 12 (29%) perceived low personal achievement. None of the residents experienced burnout with high levels of both emotional exhaustion and depersonalization and a low level of personal achievement. However, residents who met 2 of 3 criteria of burnout were able to provide information related to burnout.

The Postgraduate Hospital Educational Environmental Measure (PHEEM) revealed that most of the residents (88%) had a positive perception of their role autonomy. For the perception of teaching, 51% gave scores for teaching between 31 and 45 indicating that the teaching activities might need some improvement. For the social support dimension, 85% thought that there were more pros than cons. The average score of PHEEM was 112.7 (SD = 11.2, range 79–134), indicating a rather positive educational environment, but with room for improvement.

Only 5 (out of 41participants, 12%) reported a good quality of life, while the majority of residents (88%) reported a moderate level of overall work-related quality of life. About 10% of residents reported a low level of employee engagement, poor home-work interface and low general well-being. More than 50% (56%) of residents experienced moderate levels of stress at work and 68% had a negative work-family interface. Also, 54% of the residents were fully satisfied with the work conditions.

### Factors associated with burnout level

There were no statistically significant association between any burnout subscales and age, gender, marital status age, gender, year of training, marital status, responsibility for the family’s or own financial burden, sleep hours per night, study time hours per week and non-academic leisure time hours per week (data not shown). Work-related quality of life had a moderate correlation with emotional exhaustion (*r *= 0.401, *p* = 0.009). Emotional exhaustion, depersonalization, and personal accomplishment were not associated with the overall educational climate or residents’ perception of social support, teaching and autonomy. Total work-related quality of life had a significant small negative correlation with emotional exhaustion (*r* = − 0.401, *p* = 0.009) and with depersonalization (r = − 0.332, *p* = 0.034). Analysis of the association between the subscales of MBI and the work-related quality of life indicated a slight to moderate negative relationship between the home-work interface, general well-being, working conditions and emotional exhaustion. In addition, employee engagement, general well-being and working conditions had a slightly negative correlation with depersonalization. Also, general well-being had a moderate positive association with personal accomplishment.

### Educational climate and work-related quality of life

The educational climate and work-related quality of life were strongly related (*r* = 0.678, *p* < 0.001). The analysis of the correlations between the subscales of educational environment and work-related quality of life showed significance for multiple subscales. Perception of role autonomy had a large effect on employee engagement (*r* = 0.611, *p* < 0.001), general well-being (*r* = 0.593, *p* < 0.001) and working conditions (*r* = 0.617, *p* < 0.001), with a moderate relationship with control at work (*r* = 0.38, *p* = 0.014). Perception of teaching showed a strong relationship with employee engagement (*r* = 0.713, *p* < 0.001) and had moderate effect on control at work, general well-being, job and career satisfaction and working conditions. Perception of social support showed a significant correlation with all the subscales of work-related quality of life, except stress at work. This could indicate that QOL was affected by social support Table 1.

**Table 1:** Pearson correlation coefficients between educational environment and work-related quality of life

	Perceptions of role autonomy	Perceptions of teaching	Perceptions of social support
Employee engagement	0.611^a^	0.713^a^	0.579^a^
Control at work	0.380^b^	0.604^a^	0.469^a^
Home-work interface	0.390^b^	0.198	0.394^b^
General well-being	0.593^a^	0.408^a^	0.659^a^
Job and career satisfaction	0.261	0.510^a^	0.320^b^
Working conditions	0.617^a^	0.468^a^	0.629^a^
Stress at work	−0.094	−0.357^b^	−0.097

^a^ Pearson correlation is significant at the 0.01 level (2-tailed)

^b^ Pearson correlation is significant at the 0.05 level (2-tailed)

### Qualitative part, individual interviews

Six out of the seven residents who met the inclusion criteria of scores on burnout subscales participated in the interviews. Three were first year residents and one was a third-year resident; one participant was male. Factors in educational climate and work-related quality of life were explored in depth to find a relationship with risk of burnout. The following themes which residents perceived to relate to risk of burnout emerged from the qualitative data analysis of the individual interviews:

- Educational climate
  1. Inappropriate tasks

Residents reported execution of undesired tasks such as unnecessary or useless work which contributed to exhaustion.

*“When I rotated to a busier hospital outside my institute, I felt less exhausted, even though the number of patients under my responsibility increased. The unnecessary paper work and rule restrictions in that hospital were less than in my institute.” (Female, 3rd year resident)*

2. Teachers, teaching styles, and role as a teacher

Residents reported stress during various learning activities such as in a conference, or during ward rounds. The stress, in residents’ perception, came from, teacher’s styles, and unsafe atmosphere of some learning activities.

Constructive feedback from teachers is crucial for learners to achieve personal or teaching programme-related objectives. Residents prefer teachers who do not to display aggressive verbal communication or give negative emotional responses in public.

*“I felt pressure in the morning conference as I did not have sufficient time to prepare my case report. Sometimes I can’t answer the question, but the teacher still keeps asking more and more questions. I have nothing further to say and I feel stressed.” (Female, 3rd year resident)*

Residents’ roles as teachers to medical students was perceived as one of the stress burdens as they were expected to spend extra time to teach. Also, evaluation of their teaching at the end of rotation caused them stress and lowers their motivation to guide younger colleagues.

3. Knowledge insecurity to perform tasks

Although senior residents and attending staff were available to provide support, they sometimes felt insecure in their knowledge and felt a gap regarding patient care and consequently lacked the confidence to teach the students.

*“I can’t understand and catch up with the conversations during ward rounds with the Senior Resident and supervisors. I feel that I have insufficient knowledge.” (Female, 1st year resident)*

- Work-related quality of life
  1. The time dimension

Residents reported that extra personal time, in addition to duty hours, spent on ward rounds or other work related activities contributed to burnout.

“When I have limited personal time, I feel exhausted. I cannot remember things and this decreases my learning motivation.” (Female, 1st year resident)

2. Life crisis during training and home-work unbalance

Limitation of personal time could have an effect on the personal life of residents, especially when critical life events occurred in their private lives.

*“Before my mother passed away, I had no time to say goodbye to her. I spent my time taking care of other children instead of talking to my mother when she could communicate with me.” (Female, 2nd year resident)*

3. Physical environment and facilities

Some residents perceived the need for more and better facilities such as more dormitories and a better quality of available restaurants after office hours, instead of fast food and convenience stores. It was seen as is a way to pursuit wellbeing. Also, residents want more dormitories in the hospital, in order to create more time in the hospital in the already busy mornings.

*“I don’t live inside the hospital, I have to travel from the hospital to my home every day. I always feel exhausted.” (Female, 2nd year resident) “It would be nice to have a place to stay near the hospital, so I don’t have to waste my time in the traffic.” (Female, 3rd year resident)*

4. Role and work allocation clarity

Some work was not clearly allocated, so unexpected workload or repetitive work occurred.

“*New patients arrive on the ward at the beginning of night shifts around 5pm. Sometimes the Chief Resident assigns the resident on the ward to assess and care for the patient, instead of the resident on the night shift. Thus, the resident on the ward finishes work late and the resident on the night shift also cares for the patient simultaneously.” (Female, 1st year resident)*

In summary, the qualitative part explicated some important factors in the educational climate, such as inappropriate tasks, teaching styles and also work-related quality of life in pediatric residents’ perception, such as spending extra personal time on work-related activities, and life crisis which relate to risk of burnout.

## Discussion

In this study, we examined the prevalence of burnout among pediatric residents as well as the correlation between burnout subscales, personal characteristics, related factors in the educational climate and work-related quality of life in a non-Western context. Although, none of the pediatric residents had high burnout levels in *all three* domains, seven (17%) showed high levels in two out of three conditions studied (emotional exhaustion, level of depersonalization and perceived low personal accomplishment). Results from the survey showed that emotional exhaustion and educational climate were correlated with work-related quality of life. In the interviews, the main themes related to burnout were inappropriate tasks, teachers and teaching styles, the perception of knowledge insecurity relating to task performance, time dimensions, life crisis during training, role expectations and work allocation clarity, and facilities such as accommodation.

### Prevalence of burnout among pediatric residents

A striking finding of the quantitative study was that none of pediatric residents had high burnout levels in all three domains. The explanation of this difference could be the diversity of educational climates between institutions or the social desirability, as our study was confidential but not anonymous. However, seven of the participants in our study with warning signals regarding burnout, reported high scores on two out of the three factors: high emotional exhaustion, high depersonalisation and low personal accomplishment. A correlation between burnout subscales, educational climate and work-related quality of life among pediatric residents in the previous studies, was confirmed in our study [10–14, 20–23].

### Factors associated with burnout level

Another finding of our study was a correlation between total quality of life and emotional exhaustion. The characteristics of residents in our study demonstrated no relationship with any of the subscales of the burnout level. This is in line with previous findings Llera and Durante that demographic factors do not relate to at-risk residents [20]. However, this contrasts with findings in a study by Shanafelt et al. who identified being older and being married were related to a lower risk for burnout [27]. These differences might be explained from the context of Thai pediatric residents who are almost all of similar age, gender (female) and marital status (single).

Moreover, the burnout subscales level of pediatric residents was not related to the overall medical educational environment. This is in line with the study of Meriläinenaetal. that reported no relationship between the teaching-learning model of environment and burnout [28]. Alternatively, Dyrbye et al. discovered that some learning environment factors were associated with medical students’ burnout, but these factors were not included in the PHEEM questionnaire [9]. In Western countries, factors contributing to burnout were job satisfaction, stress and control at work, working conditions, and home-work interface [13], while this study added in a non-Western setting, the importance of inappropriate tasks, teachers and teaching styles (including unsafe environment), the perception of knowledge insecurity to perform some tasks, time dimensions, and life crisis occurring during training. These findings thus underscore that differences in factors contributing to burnout exist between different cultures.

### Educational environment and work-related quality of life

Noteworthy is that a strong association between educational environment and work-related quality of life was found. A learning climate designed to promote resident’s autonomy might benefit resident’s work-related quality of life. Perception of a good teaching climate showed a positive relationship with employee engagement, a moderate association with control at work, general well-being, job and career satisfaction and working conditions, and a negative correlation for stress at work. In conclusion, educational climate had effects not only on residents wellbeing but also on stress at work. Thai clinical teachers and curriculum developers, therefore, need to pay more attention to the effect of educational climate rather than focusing on students learning only. Social support showed a significant correlation with all subscales of work related quality of life, except for stress at work. One possible interpretation is that social support in Thai context could improve the residents’ quality of life but is not likely to reduce the stress level at work. Social support in the workplace in the Thai context seems to be lacking at this moment. Nevertheless, social support in the Thai context should be considered as important for residents wellbeing and would, consequently, needs more attention. Further evidence for this is found in a study among internal medicine and pediatric residents in U.S., where loneliness was associated with burnout, sense of personal accomplishment was associated with greater network centrality [29], and interpersonal relationship was related to burnout [10].

The PHEEM questionnaire did not provide detailed information regarding burnout or stress at work and the improvement of the educational environment. The only item of PHEEM with an average score lower than two out of five in the social support subscale was ‘there are adequate catering facilities when I am on call’. However, the qualitative study identified some details of work and workloads, roles and relationships with other professions, learning atmosphere, research conduction, teachers’ responses, accommodation and restaurants, not included in the PHEEM. These issues are especially important for the residents training programme in Thailand.

A study by Tsai et al. used a new instrument to investigate the relationship between the clinical learning environment and mental distress among post graduate medical students in Taiwan, because of the socio-culture differences between Taiwan and Western countries [30]. Unfortunately, this instrument was not validated or tested in other socio-cultural contexts, while PHEEM is currently used in many countries such as UK, Canada, Ireland, China, Indonesia, Malaysia, Norway, Sweden, Brazil, the West Indies and the Yemen [24]. The PHEEM, assessing the educational environment, might not be the most suitable tool to assess the stress and resident training environment context in Thailand. Therefore, a new, more valid, questionnaire should be developed to assess the educational environment in the context of non-Western medical education.

The qualitative data also indicated that changes to the educational environment are necessary to lower the risk of burnout. For example, a medical service system that promotes collaboration, is user friendly and decreases unnecessary work, might facilitate the work of residents and lower the levels of exhaustion. Moreover, a predictable or routine time schedule for working and learning on the wards was preferred by the residents. The teaching schedule should fit with the flow of work during the day and also not disturb residents’ personal time. This finding was similar to U.S. pediatric residents reporting that time demands contributed to burnout [10]. In addition, the role and work allocation should be clarified and properly scheduled to fit with the learning process and minimize learning disturbances at the workplace. Lastly, this study suggested that considering adopting different teaching styles and methods such as constructive feedback, investing time and effort in interventions contributing to a safe learning climate and paying attention to a faculty mistreatment which could contribute to medical students burnout. Further studies focusing on teaching styles, safe learning climate and mistreatment in a non-Western context are needed. Also the provision of improved faculty development programmes might enhance teaching, assessment and curriculum design and might require institutional policies supporting the programme [31].

### Strengths and limitations of the study

The strength of this study is the mixed method design, combining quantitative and qualitative methodologies, which facilitates in-depth information gathering on the related factors of burnout and also exploration of other factors included in the quantitative part. This study also has limitation such as the small sample size, setting limitations to multivariate analysis. Also, the relationship between researchers and participants requires consideration. Although participants were informed that the data was confidential, they might not be comfortable to reveal negative information or may have given socially and culturally desirable answers. Moreover, this exploratory study was conducted in only one hospital, which might limit its generalizability to other settings. Further studies will be needed.

## Conclusion

Although a relation was found between subscales of burnout, quality of life and educational climate, this mixed methods study did not show substantial burnout among pediatric residents in the Thai context.

The factors of the educational environment that were perceived to require improvement in order to reduce the risk of burnout were: minimization of unnecessary or duplicated workloads, time schedule arrangements to avoid extension of regular duty hours, and the clarity of role expectations, work allocation, perceptions of teacher roles, institution of a faculty development programme, and improvement of the facilities and the infra-structure such as accommodation. These factors should consequently be considered and addressed in the curriculum and clinical workplace of pediatric residents in training**.**

## Data Availability

Not applicable: data and materials are confidential.

## Abbreviations

AAP: American Academy of Pediatrics
CAW: Control at work
EET: Employee engagement
GWB: General well-being
HWI: Home-work interface
IRB: Institutional Review Board
JCS: Job and career satisfaction
JD: Job demands
JD-R: Job demands-resources
MBI: Maslach Burnout Inventory
PHEEM: Postgraduate Hospital Educational Environmental Measure
R: Resources
SAW: Stress at work
WCS: Working conditions
WRQoL: Work-Related Quality of Life scale
