# Correction to: Exploring burnout and the association with the educational climate in pediatric residents in Thailand

**DOI:** 10.1186/s12909-019-1723-7

**Published:** 2019-08-01

**Authors:** Pongtong Puranitee, Fred F. C. J. Stevens, Samart Pakakasama, Adisak Plitponkarnpim, Sakda Arj-Ong Vallibhakara, Jamiu O. Busari, Sylvia Heeneman, Walther N. K. A. van Mook

**Affiliations:** 1 Faculty of Medicine Ramathibodi Hospital, Mahidol University, 270 Rama VI Road, Rajthevi, Bangkok 10400 Thailand; 2 Department of Educational Development & Research, Faculty of Health, Medicine & Life Sciences (FHML), Maastricht University (UM), Maastricht, The Netherlands; 3 Section for Clinical Epidemiology and Biostatistics, Faculty of Medicine Ramathibodi Hospital, Mahidol University, 270 Rama VI Road, Rajthevi, Bangkok 10400 Thailand; 4 Child Safety Promotion and Injury Prevention and Research Center, Faculty of Medicine Ramathibodi Hospital, Mahidol University, 270 Rama VI Road, Rajthevi, Bangkok 10400 Thailand; 5 Department of Pathology, Faculty of Health, Medicine & Life Sciences (FHML), School of Health Profession Education, Maastricht University (UM), Maastricht, The Netherlands; 6 Department of Intensive Care Medicine, Maastricht University Medical Centre, Maastricht, The Netherlands

**Correction to: Puranitee et al. BMC Medical Education (2019) 19:245**

**https://doi.org/10.1186/s12909-019-1687-7**

Following publication of the original article [1], the author reported that the given name and family name of Sakda Arj-Ong Vallibhakara:

Incorrect tagging of name in the original article:

Given name: Sakda

Family Name: Arj-OngVallibhakara

Correct tagging of name:

Given name: Sakda

Given name: Arj-Ong

Family Name: Vallibhakara

The original article has been corrected.
